# Single-Position Minimally Invasive Retropleural Asymmetric Vertebral Column Resection and Percutaneous Pedicle Screw Fixation in the Lateral Position for Congenital Kyphoscoliosis

**DOI:** 10.3390/jcm15155861

**Published:** 2026-07-27

**Authors:** Piotr Kowalski, Gergely Bodon, Michael A. Galgano, Justyna Walczak, Michał Grabala, Krzysztof Zakrzewski, Paweł Grabala

**Affiliations:** 1 Department of Neurosurgery, Regional Specialized Hospital, ul. Dekerta 1, 66-400 Gorzow, Poland; pkowal72@gmail.com; 2 Department of Orthopaedic Surgery, Klinikum Esslingen, 73730 Esslingen am Neckar, Germany; g.bodon@klinikum-esslingen.de; 3 Department of Neurosurgery, University of North Carolina, Chapel Hill, NC 27516, USA; mgalgano@email.unc.edu; 4 Department of Neurosurgery, Polish-Mother’s Memorial Hospital Research Institute, Rzgowska 281/289, 93-338 Lodz, Polandkrzysztof.zakrzewski@iczmp.edu.pl (K.Z.); 5 2nd Clinical Department of General and Gastroenterogical Surgery, Medical University of Bialystok Clinical Hospital, ul. M. Skłodowskiej-Curie 24a, 15-276 Bialystok, Poland; michal@grabala.pl

**Keywords:** congenital kyphoscoliosis, hemivertebra resection, minimally invasive spine surgery, lateral retropleural approach, thoracic deformity, percutaneous pedicle screws, rigid spinal deformity, asymmetric vertebral column resection, posterior vertebral column resection

## Abstract

**Background:** Congenital thoracic kyphoscoliosis caused by vertebral malformations is a challenging condition that may progress during skeletal growth, leading to spinal imbalance, pain, cosmetic deformity, and neurological compromise. Conventional correction often requires extensive anterior, posterior, or combined approaches associated with substantial surgical morbidity. We describe a single-position surgical technique combining a lateral retropleural asymmetric vertebral resection with percutaneous posterior instrumentation performed entirely in the lateral decubitus position. **Methods:** A 15-year-old boy with progressive congenital thoracic kyphoscoliosis secondary to a T10 butterfly vertebra underwent surgical correction after failure of conservative treatment. The procedure was performed entirely in the left lateral decubitus position under multimodal intraoperative neurophysiological monitoring. Bilateral percutaneous pedicle screws were inserted from T7 to L1 under fluoroscopic guidance without repositioning the patient. A muscle-sparing lateral retropleural approach was then used to perform T10 asymmetric vertebral column resection, anterior column reconstruction with an expandable cage, and definitive deformity correction using posterior rod compression. The technical rationale, operative workflow, and reconstruction strategy are described. **Results:** The procedure was completed without intraoperative neurological deterioration or the need for patient repositioning. Postoperative imaging demonstrated satisfactory restoration of coronal and sagittal alignment, appropriate implant positioning, and spinal canal decompression. The patient experienced marked improvement in pain, shoulder balance, rib hump deformity, and overall posture while maintaining normal neurological function. The main thoracic curve improved from 32° to 6°, thoracic kyphosis from 78° to 63°, VAS from 5 to 0, ODI from 42 to 5, and SRS-22R from 3.85 to 4.85. Solid fusion was confirmed at 3 years. A postoperative pneumothorax, attributed to pleural violation during exposure, represented the only complication and resolved completely following pleural drainage. At 36-month follow-up, radiographs and computed tomography confirmed maintenance of deformity correction, stable instrumentation, and solid anterior and posterior fusion without implant failure or loss of correction. **Conclusions:** Single-position lateral retropleural asymmetric vertebral resection combined with percutaneous pedicle screw fixation is a technically feasible option for selected patients with congenital thoracic kyphoscoliosis. Avoiding intraoperative repositioning while combining anterior reconstruction and posterior stabilization through a reduced-access retropleural approach may simplify the surgical workflow and minimize soft-tissue disruption without compromising deformity correction. Further clinical experience is required to establish its reproducibility and comparative advantages.

## 1. Introduction

Congenital spinal deformities are uncommon but clinically significant disorders resulting from abnormalities of vertebral formation, segmentation, or both during embryonic development [1,2]. Congenital kyphoscoliosis caused by vertebral anomalies such as hemivertebrae, butterfly vertebrae, wedge vertebrae, or unilateral segmentation defects frequently progresses during skeletal growth and may lead to progressive sagittal and coronal imbalance, chronic pain, thoracic cage deformity, cardiopulmonary compromise, and, in advanced cases, spinal cord compression with neurological deterioration [1,2,3,4,5,6,7,8,9,10,11]. Because these deformities are structural rather than functional, conservative treatment, including physiotherapy, postural rehabilitation, and bracing, has limited ability to prevent curve progression [1,3,6,11,12]. Consequently, progressive congenital kyphoscoliosis often requires surgical correction to restore spinal alignment, prevent neurological compromise, and improve functional and cosmetic outcomes [1,5,6,12].

Surgical management of congenital thoracic kyphoscoliosis remains technically demanding. Depending on deformity severity and vertebral morphology, treatment may involve posterior-only correction, combined anterior–posterior procedures, or vertebral column resection [8,10,11,12,13,14]. Although these techniques can achieve satisfactory deformity correction, they frequently require extensive soft-tissue dissection, prolonged operative time, and substantial blood loss and are associated with postoperative pain and pulmonary complications. In particular, conventional anterior thoracic approaches often require thoracotomy with significant chest wall disruption and pleural manipulation, contributing to increased perioperative morbidity and delayed recovery [3,6,15,16,17,18].

The lateral retropleural approach (LRPA) has emerged as an alternative access corridor to the thoracic spine that preserves the extrapleural plane and avoids formal thoracotomy while providing direct exposure of the vertebral body [19,20,21,22]. When combined with endoscopic illumination, microscopic visualization, and percutaneous pedicle screw fixation, this approach may reduce muscle dissection, limit surgical exposure, and facilitate anterior column reconstruction through a smaller operative corridor while maintaining the biomechanical principles of deformity correction [20,21,23]. Nevertheless, experience with this technique for congenital thoracic deformity remains extremely limited, particularly when both anterior reconstruction and posterior instrumentation are performed entirely in the lateral decubitus position.

The principal technical challenge is not the retropleural exposure itself, but the integration of vertebral resection, anterior column reconstruction, and bilateral percutaneous pedicle screw fixation into a single-position workflow without intraoperative repositioning. This strategy requires careful preoperative planning, precise fluoroscopic orientation, reproducible patient positioning, and a well-defined sequence of correction maneuvers, all of which remain insufficiently described in the current literature [19,20,21,22]. Accordingly, the purpose of this technical note is to describe the operative workflow, technical rationale, surgical pearls, and reconstruction strategy of a single-position lateral retropleural hemicorpectomy combined with bilateral percutaneous pedicle screw fixation for congenital thoracic kyphoscoliosis. Using an illustrative case, we highlight the technical considerations, indications, potential pitfalls, and early clinical and radiographic outcomes associated with this procedure.

## 2. Methods/Case Presentation

### 2.1. Case Presentation

**Figure 1:**
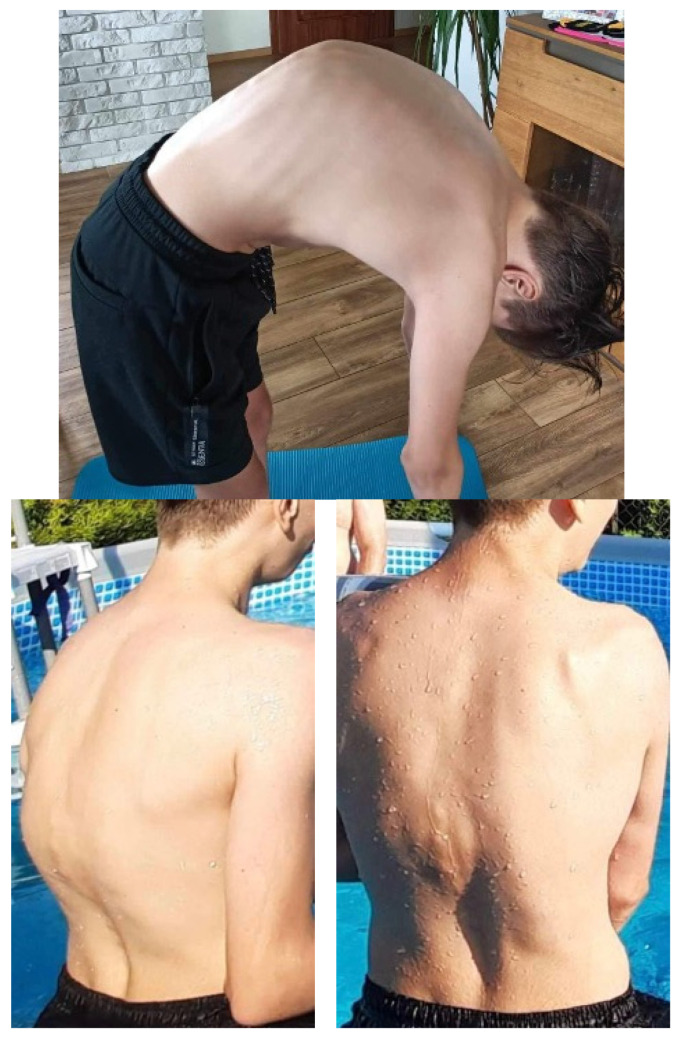
Clinical pictures of a 15-year-old boy with a progressive congenital spinal deformity that had first been detected about 6 months before consultation.

A 15-year-old boy was referred to our institution because of progressive congenital thoracic kyphoscoliosis associated with worsening thoracic hyperkyphosis, increasing right-sided rib prominence, shoulder imbalance, and persistent mechanical thoracic pain. Despite prolonged conservative treatment consisting of physiotherapy, postural rehabilitation, and regular orthopedic follow-up, both the clinical deformity and radiographic parameters continued to deteriorate. There was no history of previous spinal surgery, trauma, neuromuscular disease, or connective tissue disorder. Neurological examination demonstrated normal motor and sensory function, symmetrical deep tendon reflexes, and no evidence of myelopathy. Clinical assessment revealed marked thoracic hyperkyphosis, trunk asymmetry, and a prominent right rib hump (Figure 1).

**Figure 2:**
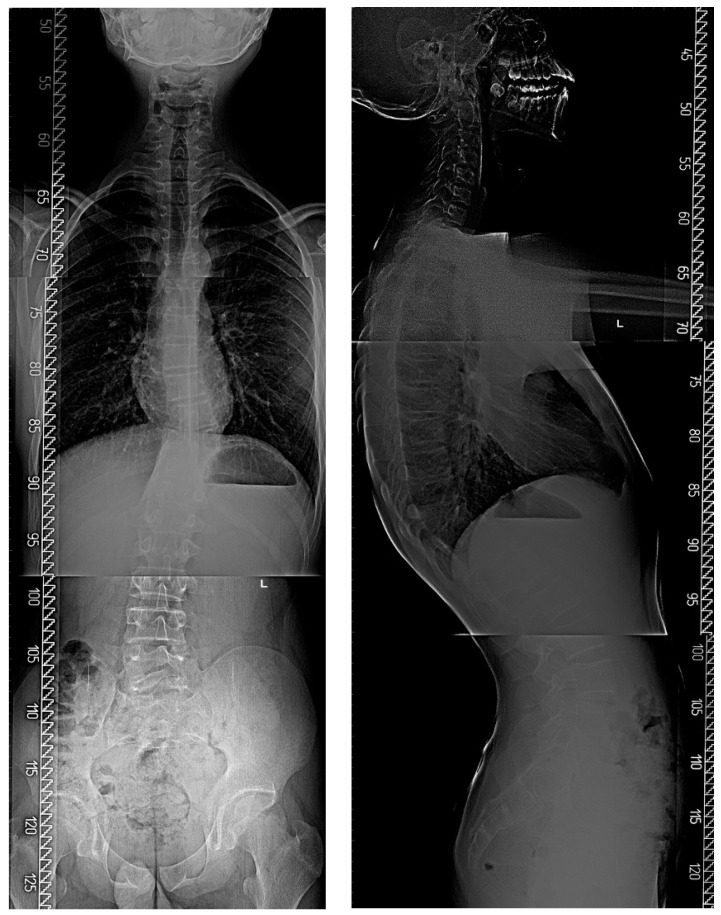
Standing radiographs of the entire spine of a 15-year-old boy with a progressive congenital spinal deformity.

**Figure 3:**
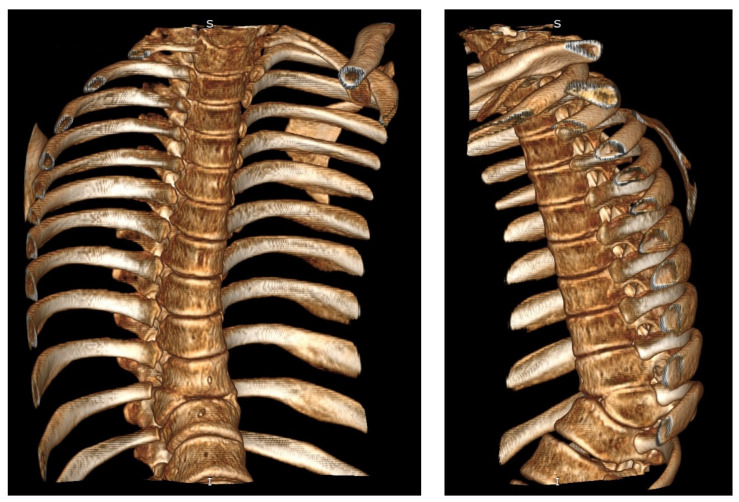
3D computed tomography reconstructions shows the butterfly vertebra of Th10 driving the kyphoscoliotic deformity in a 15-year-old boy.

Standing full-spine radiographs demonstrated congenital thoracic kyphoscoliosis associated with a T10 butterfly vertebra resulting in progressive coronal and sagittal imbalance (Figure 2). Computed tomography confirmed the complex vertebral anatomy and demonstrated shortening of the anterior spinal column caused by the malformed vertebra (Figure 3). This led to shortening of the anterior column, causing the kyphotic deformity. The asymmetric growth led to the scoliosis due to shortening of the right side of the spine.

**Figure 4:**
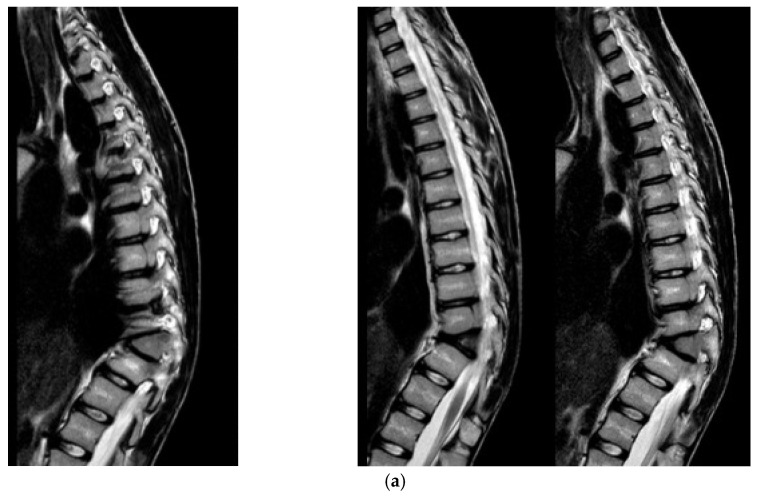

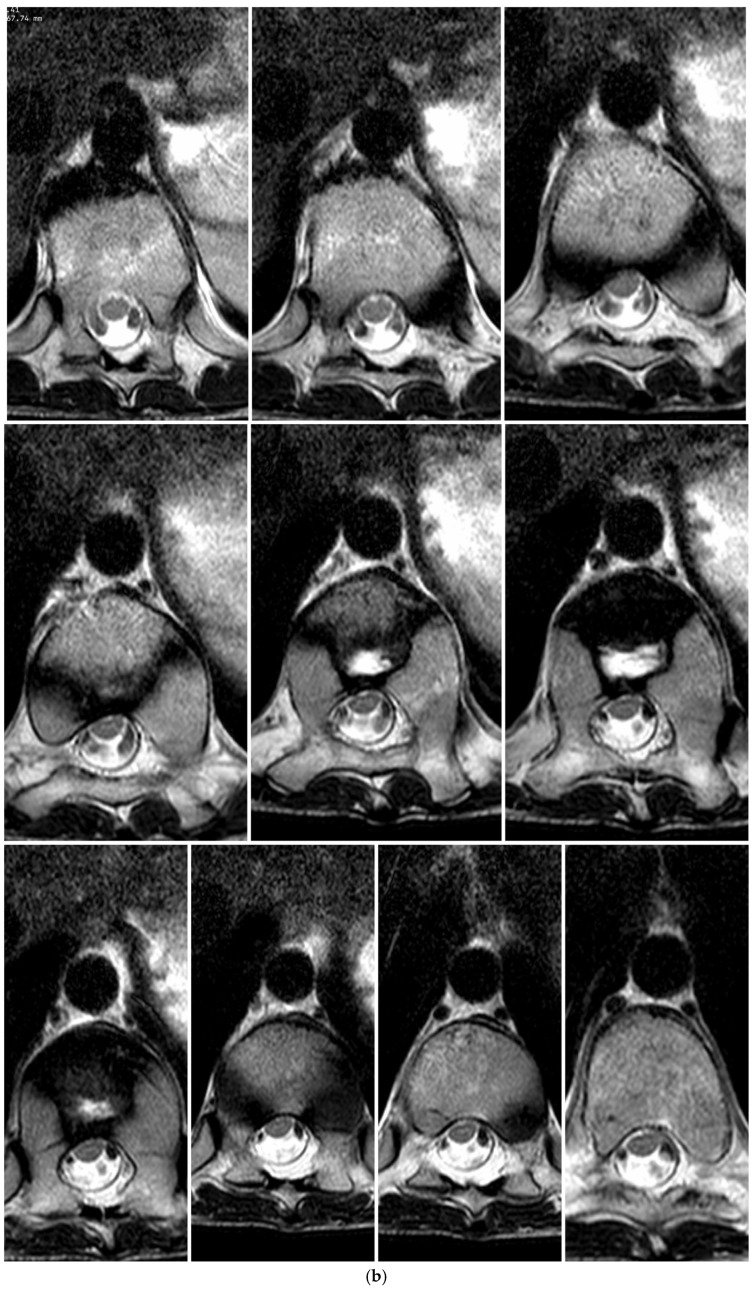
(**a**) Magnetic resonance imaging of a 15-year-old boy revealed progressive narrowing of the thoracic spinal canal secondary to deformity progression due to a butterfly vertebra. (**b**) Sequential T2-weighted magnetic resonance imaging (MRI) scans demonstrating the pathological vertebra. Consecutive axial images progress caudally through the affected vertebral level, illustrating the morphology of the vertebral anomaly and its relationship to the spinal canal.

Magnetic resonance imaging revealed progressive narrowing of the thoracic spinal canal without neurological deficit, indicating an increasing risk of future spinal cord compromise (Figure 4a,b).

Because of documented deformity progression, persistent symptoms, progressive spinal canal narrowing, and the anticipated risk of neurological deterioration, surgical correction was recommended. The surgical objectives were restoration of sagittal and coronal alignment, vertebral resection with anterior column reconstruction, spinal stabilization, and prevention of future neurological compromise. For this reason, a thorough anterior release between the Th9 and Th11 vertebrae, asymmetric vertebral column resection of the right hemivertebra (butterfly vertebra) and lengthening of the right side was planned.

### 2.2. Surgical Technique

#### 2.2.1. Surgical Concept

The rationale behind this procedure was to combine anterior vertebral resection, anterior column reconstruction, and definitive posterior stabilization into a single-position operation without intraoperative repositioning. Unlike conventional combined anterior–posterior surgery, which requires repositioning from the lateral to the prone position, this technique enables completion of the entire procedure while maintaining the patient in the left lateral decubitus position. Eliminating repositioning reduces interruptions in the surgical workflow, avoids repeated patient preparation and redraping, decreases the risk of contamination, and allows continuous maintenance of neuromonitoring throughout the operation. The retropleural approach was selected to obtain direct lateral access to the vertebral body while preserving the extrapleural plane whenever possible and avoiding a formal thoracotomy. Posterior stabilization was performed percutaneously to minimize posterior muscle dissection while providing stable fixation for deformity correction and anterior column reconstruction.

#### 2.2.2. Preoperative Planning

Comprehensive preoperative planning was performed using standing radiographs, computed tomography, and magnetic resonance imaging. The imaging studies enabled detailed analysis of vertebral anatomy, pedicle morphology, spinal canal dimensions, and the relationship of the malformed vertebra to adjacent neural and vascular structures. The surgical strategy was designed to achieve deformity correction through a combined minimally invasive anterior and posterior stabilization procedure performed without repositioning the patient. A lateral retropleural approach was selected to minimize thoracic wall trauma and avoid formal thoracotomy. Continuous intraoperative neurophysiological monitoring was planned throughout the procedure, including somatosensory evoked potentials and motor evoked potentials, to minimize the risk of neurological injury during deformity correction and vertebral resection. Particular attention was paid to determining the exact skin incision, fluoroscopic projection, pedicle trajectories, cage dimensions, and sequence of deformity correction before surgery.

#### 2.2.3. Patient Positioning

**Figure 5:**
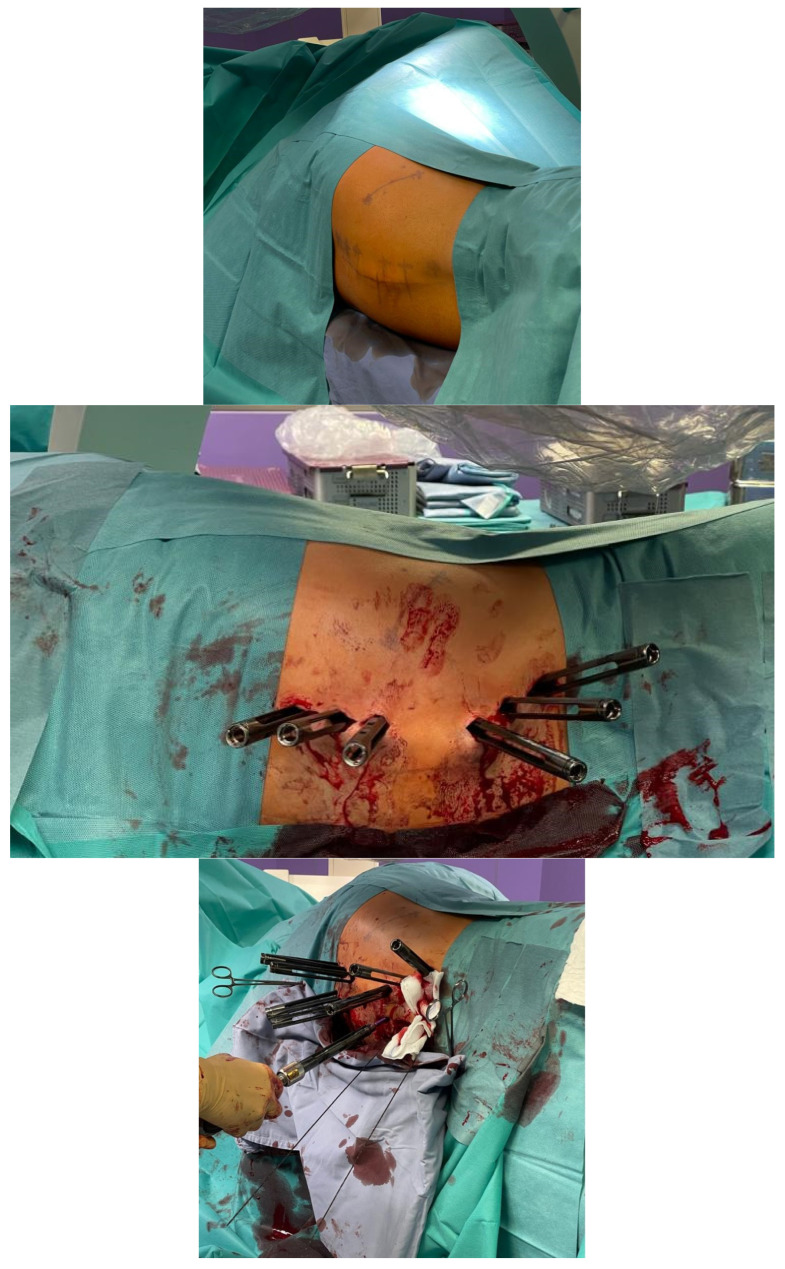
Intraoperative fluoroscopic-guided percutaneous pedicle screw fixation at T7–L1 performed using minimally invasive spinal surgery techniques.

The patient was positioned in a true left lateral decubitus position on a radiolucent operating table. An axillary roll was placed beneath the dependent hemithorax to prevent brachial plexus compression, while additional padding protected all pressure points. The operating table was adjusted to achieve a true lateral fluoroscopic projection of T9–T11 before skin preparation. Minor table rotation was subsequently used during the procedure to optimize fluoroscopic visualization during pedicle screw insertion and cage implantation. The entire thoracolumbar region extending from the posterior midline to the anterior axillary line was prepared and draped, allowing simultaneous access to both the lateral thoracic approach and the posterior percutaneous screw entry points without repositioning. Throughout the operation, the patient remained in the same position, permitting uninterrupted neuromonitoring and eliminating the need for repeated positioning, reprepping, or redraping. The thoracic and lumbar regions were prepared and draped in a sterile fashion (as shown in Figure 5). The lateral position was maintained throughout the entire operation, eliminating the need for intraoperative repositioning.

#### 2.2.4. Percutaneous Pedicle Screw Placement

The first stage of the operation involved minimally invasive posterior percutaneous pedicle screw fixation. Under fluoroscopic guidance, bilateral pedicle screws were inserted percutaneously at T7, T8, T9, T11, T12, and L1 according to the technique described by Bodon et al. [24]. Pedicle screw fixation was achieved using the Reline^®^ posterior fixation system, a thoracolumbar pedicle screw–rod construct designed for spinal stabilization and fusion procedures (NuVasive, currently part of Globus Medical, 2560 General Armistead Avenue, Audubon, PA 19403, USA). Small paramedian skin incisions were created corresponding to the planned screw trajectories. Sequential dilation and cannulated instrumentation techniques were used in accordance with standard MISS principles. Guidewires were advanced under fluoroscopic visualization, followed by pedicle preparation and screw insertion (Figure 5). Particular attention was paid to accurate pedicle trajectory because congenital deformities may significantly distort normal anatomical landmarks. Fluoroscopic control in anteroposterior and lateral projections was repeatedly used to verify appropriate implant positioning. Following screw placement, temporary rods were prepared, but definitive correction was deferred until completion of vertebral resection and anterior reconstruction [24,25]. Particular technical attention was required during insertion of the pedicle screws on the dependent side because the operating table limited instrument angulation. Slight table rotation together with oblique fluoroscopic projections allowed safe pedicle access while preserving the minimally disruptive percutaneous technique. Screw insertion was performed sequentially from cranial to caudal levels before the lateral exposure to maintain stable reference anatomy and avoid interference between the posterior instrumentation and the lateral working corridor.

#### 2.2.5. Lateral Retropleural Approach

##### Patient Orientation and Fluoroscopic Confirmation

Following completion of bilateral percutaneous pedicle screw insertion, the patient’s position was carefully re-evaluated before beginning the lateral approach. The operating table was gently tilted until the T9–T11 vertebral bodies were visualized in a true lateral fluoroscopic projection. Achieving a true lateral orientation is essential because even minor rotational malalignment compromises the accuracy of the retropleural exposure and subsequent cage implantation. The planned skin incision was determined under fluoroscopic guidance by identifying the T8–T9 and T11–T12 intervertebral disc spaces. A straight oblique line connecting the posterior aspect of the cranial disc and the anterior aspect of the caudal disc was marked on the skin, thereby defining the optimal working corridor centered over the pathological T10 vertebra.

##### Muscle-Sparing Retropleural Exposure

**Figure 6:**
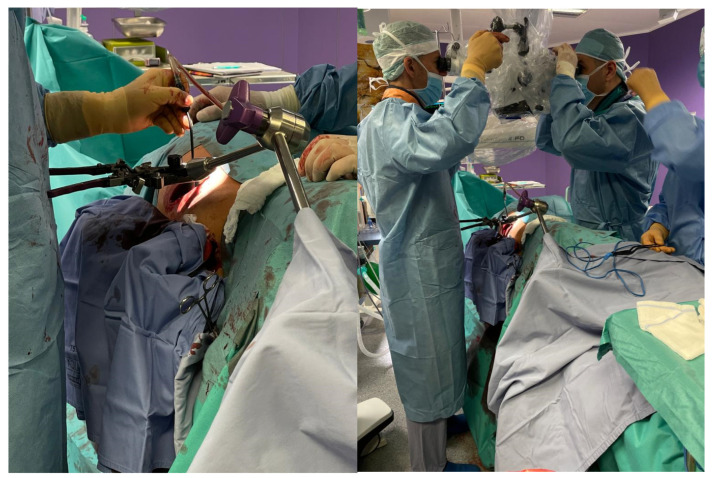
Intraoperative pictures show the thoracotomy and the lateral retractor system (MaxAccess Retractor Globus Medical). The screw towers are covered with sterile drapes (blue), and pedicle screw fixation was achieved using the Reline^®^ posterior fixation system, a thoracolumbar pedicle screw–rod construct designed for spinal stabilization and fusion procedures (NuVasive, currently part of Globus Medical, USA).

Following skin incision, the subcutaneous tissue and thoracolumbar fascia were divided. The lateral border of the latissimus dorsi muscle was identified, mobilized, and gently retracted posteriorly while preserving the muscle fibers whenever possible. The rib corresponding to the T10 vertebral level was identified fluoroscopically and exposed by subperiosteal dissection over approximately 10 cm (Figure 6). The intercostal neurovascular bundle was carefully identified and mobilized from the inferior surface of the rib. Circumferential subperiosteal dissection allowed safe resection of the rib while preserving it for later use as an autologous bone graft. The retropleural plane was subsequently developed using blunt peanut dissectors and long handheld retractors until the lateral aspect of the T10 vertebral body was reached. Particular attention was paid to preserving the extrapleural plane and minimizing pleural mobilization throughout the exposure. Although preservation of pleural integrity was intended, pleural violation remains a recognized risk of thoracic retropleural surgery, particularly in patients with congenital deformity and distorted anatomy. The three-bladed expandable retractor system (MaxAccess^®^, Globus Medical) was then inserted and centered over the pathological vertebra (Figure 6). Correct vertebral level localization was reconfirmed fluoroscopically before proceeding.

##### Vertebral Exposure and T10 Asymmetric Vertebral Column Resection

Discectomy was initially performed at the T9–T10 level, followed by meticulous endplate preparation and contralateral annular release using a Cobb elevator. The retractor was subsequently repositioned over the T10–T11 disc space, where an identical procedure was completed. Then, the malformed T10 butterfly vertebra was circumferentially exposed from the right side. The lateral vertebral cortex was identified, and using an osteotome, the vertebral body was separated from the posterior vertebral wall, approximately 4–5 mm of which was intentionally preserved and left attached to the pedicle to enhance neurological safety. The vertebral body was subsequently removed in a piecemeal fashion until complete anterior decompression had been achieved. The adjacent T9 and T11 endplates were then meticulously prepared for anterior column reconstruction.

##### Deformity Correction and Anterior Column Reconstruction

Following completion of the hemicorpectomy (an asymmetric vertebral column resection performed from the right side of the malformed T10 butterfly vertebra), definitive precontoured rods were inserted through the previously placed percutaneous pedicle screws (Figure 5). The caudal locking caps were tightened first, whereas the cranial fixation remained temporarily unlocked. Controlled reduction of the rods into the cranial screw heads produced gradual correction of the kyphotic deformity while avoiding excessive corrective forces. An expandable titanium vertebral body cage was subsequently inserted into the corpectomy defect under continuous fluoroscopic guidance. The cage was packed with morselized autologous bone harvested during the corpectomy together with bone substitute before gradual expansion. Progressive cage distraction simultaneously restored anterior column height, corrected sagittal alignment, and improved coronal balance. Final fluoroscopic assessment in both anteroposterior and lateral projections confirmed satisfactory deformity correction and implant positioning before definitive tightening of all locking caps.

##### Final Construct Assessment and Wound Closure

**Figure 7:**
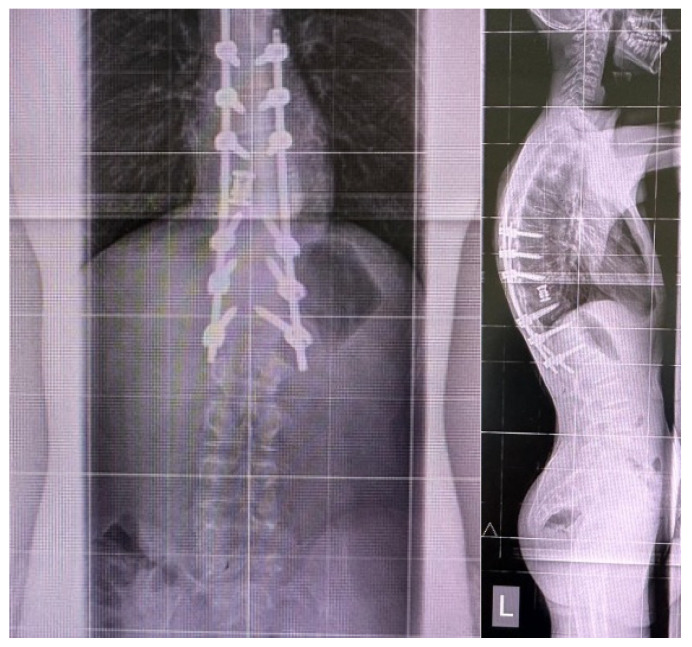
Postoperative radiograph scans demonstrating successful correction of sagittal and coronal deformity with restoration of thoracic alignment and spinal balance. Anterior vertebral body reconstruction and posterior instrumentation remained in satisfactory position with adequate spinal canal decompression.

Following definitive construct assembly, fluoroscopic imaging confirmed satisfactory restoration of sagittal and coronal alignment, together with appropriate implant positioning (Figure 7). The pedicle screw extension towers were removed (Figure 6), meticulous hemostasis was achieved, and a low-pressure suction drain was placed within the lateral operative corridor. Layered wound closure was then performed in the standard fashion. Continuous multimodal intraoperative neuromonitoring remained stable throughout vertebral resection, deformity correction, and anterior column reconstruction, with no significant changes in somatosensory or motor evoked potentials.

#### 2.2.6. Reconstruction Strategy

The reconstruction strategy was designed to simultaneously restore sagittal and coronal alignment, provide immediate circumferential spinal stability, and promote solid biological fusion while avoiding patient repositioning. Posterior stabilization was established first by percutaneous pedicle screw fixation extending from T7 to L1, providing a stable foundation for controlled deformity correction. The fixation construct intentionally spanned multiple vertebral levels above and below the pathological vertebra to distribute corrective forces over a longer lever arm and reduce mechanical stress at the apex of the deformity. This long-segment construct was considered particularly important because congenital vertebral anomalies are frequently associated with altered vertebral morphology, asymmetric loading, and increased risk of implant failure. Anterior column reconstruction was performed following T10 asymmetric vertebral column resection using an expandable titanium vertebral body cage inserted through the lateral retropleural corridor. The expandable cage allowed gradual restoration of anterior column height under continuous fluoroscopic control while simultaneously contributing to correction of both the kyphotic and scoliotic components of the deformity. Controlled cage expansion also enabled progressive load sharing between the anterior and posterior columns, thereby reducing excessive corrective forces transmitted through the posterior instrumentation alone.

Biological reconstruction relied on circumferential fusion. The expandable cage was packed with locally harvested autologous bone obtained during vertebral resection, supplemented with bone substitute to maximize the fusion surface within the anterior column. Posterior fusion was achieved through decortication of the posterior elements and placement of additional bone graft after completion of deformity correction. This combined anterior–posterior fusion strategy was intended to maximize long-term construct stability while minimizing the risk of pseudarthrosis and correction loss. The sequence of reconstruction was also considered an important technical aspect of the procedure. Posterior instrumentation was completed before vertebral resection to provide immediate spinal control throughout the operation. Following hemicorpectomy, gradual rod reduction was performed prior to definitive cage expansion, allowing controlled deformity correction while minimizing excessive stress on either the spinal cord or the instrumentation. Final locking of the construct was performed only after satisfactory restoration of alignment had been confirmed fluoroscopically in both anteroposterior and lateral projections. Overall, this reconstruction strategy combines the biomechanical advantages of circumferential stabilization with the potential benefits of a single-position muscle-sparing approach, enabling simultaneous anterior reconstruction and posterior fixation without intraoperative repositioning.

#### 2.2.7. Postoperative Management

Following surgery, the patient was transferred to the intensive care unit for overnight monitoring, with particular attention paid to neurological status, respiratory function, and hemodynamic stability. Immediate postoperative neurological examination demonstrated preserved motor and sensory function without any new neurological deficits. Routine postoperative chest radiography demonstrated a pneumothorax secondary to inadvertent pleural violation during the retropleural exposure. The patient underwent prompt tube thoracostomy, resulting in complete lung re-expansion without further pulmonary complications. The chest drain was subsequently removed after resolution of the air leak and radiographic confirmation of full pulmonary expansion. Postoperative pain was managed using a standardized multimodal analgesic protocol, allowing early mobilization beginning on the first postoperative day. Respiratory physiotherapy, incentive spirometry, and supervised rehabilitation were initiated to reduce pulmonary morbidity and facilitate functional recovery.

The patient demonstrated progressive improvement in posture, truncal balance, and ambulatory function throughout hospitalization. Clinical examination showed marked reduction of the thoracic rib hump, restoration of shoulder symmetry, and improved overall spinal alignment. The patient was discharged neurologically intact with no implant-related or wound complications. Although the retropleural approach is intended to preserve the pleural cavity, pleural violation remains a recognized intraoperative risk, particularly in patients with congenital thoracic deformities and distorted regional anatomy. When promptly recognized and appropriately managed, this complication does not appear to compromise the overall clinical outcome.

## 3. Results

### 3.1. Immediate Postoperative Outcome

The procedure was completed successfully without intraoperative neurological deterioration or implant-related complications. Postoperative radiographs and computed tomography confirmed satisfactory restoration of both sagittal and coronal spinal alignment together with appropriate positioning of the anterior expandable cage and posterior pedicle screw construct. Adequate decompression of the spinal canal was achieved, and no evidence of implant malposition or hardware loosening was identified (Figure 7).

### 3.2. Clinical Outcome

**Figure 8:**
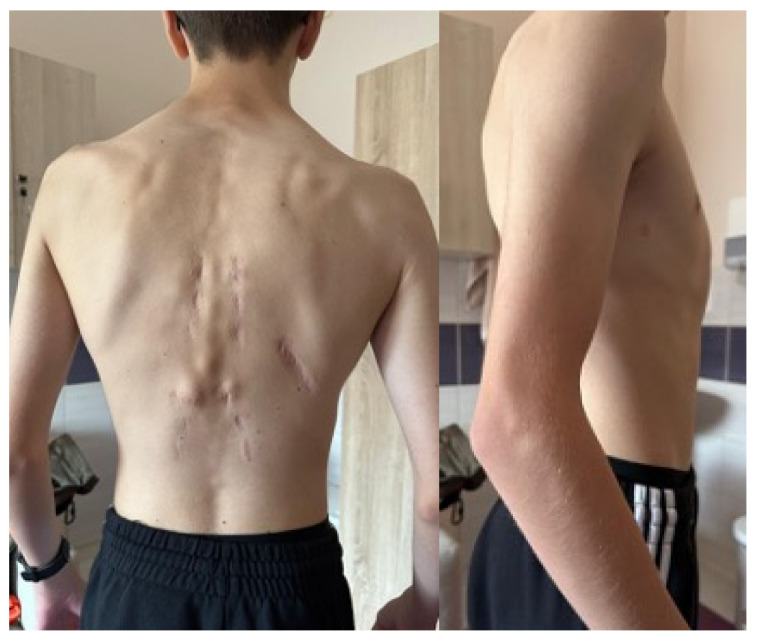
Clinical examination at 6-month follow-up demonstrating improved thoracic alignment and shoulder symmetry and reduction of rib prominence after surgical correction. The patient reported significant pain relief and satisfaction with cosmetic and functional outcomes without neurological complications.

**Figure 9:**
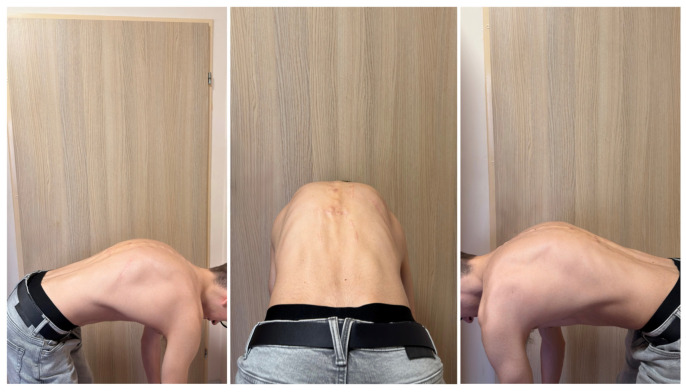

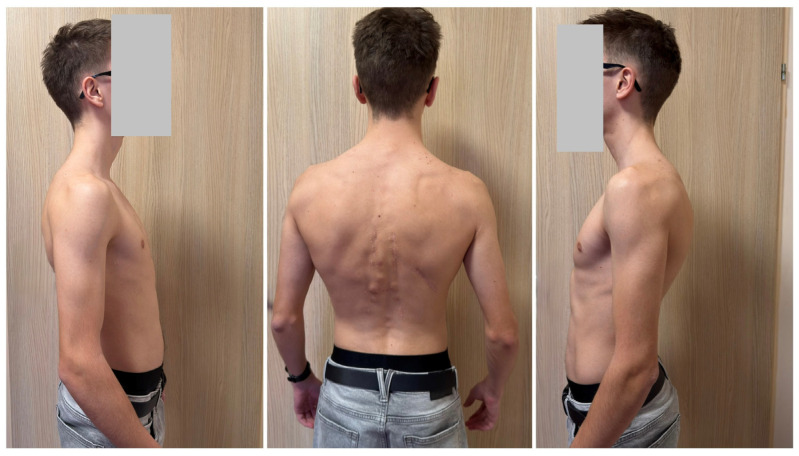
Clinical evaluation at 36-month follow-up demonstrating improved thoracic alignment and shoulder symmetry and reduction of rib prominence after surgical correction. The patient reported significant pain relief and satisfaction with cosmetic and functional outcomes without neurological complications.

The postoperative neurological examination remained normal, with preserved motor and sensory function in both lower extremities. During hospitalization, the patient experienced progressive improvement in posture, trunk balance, and ambulatory function without evidence of new neurological deficits. At the 6-month follow-up, clinical examination demonstrated marked improvement in thoracic alignment, restoration of shoulder symmetry, and substantial reduction of the right-sided rib prominence (Figure 8). The patient reported complete resolution of mechanical thoracic pain and expressed a high level of satisfaction with both the cosmetic and functional outcomes of the procedure. These improvements were maintained throughout follow-up. At 36 months, clinical assessment confirmed durable correction of the trunk deformity with preservation of shoulder balance and sustained improvement in cosmetic appearance (Figure 9). No late neurological deterioration or wound-related complications occurred.

### 3.3. Radiographic Outcome

**Figure 10:**
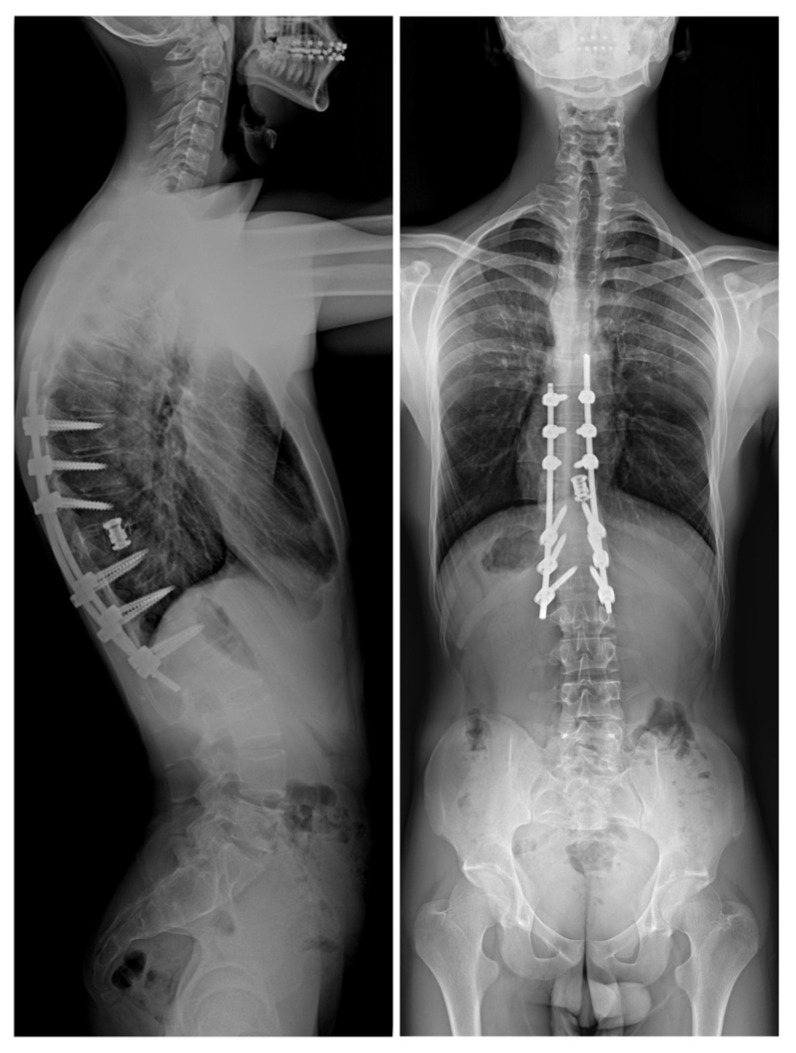
Anteroposterior (AP) and lateral (LAT) radiographs obtained 3 years after surgery, demonstrating maintained spinal correction, stable implant position, and satisfactory coronal and sagittal alignment. No evidence of hardware failure, loss of correction, or other postoperative complications was observed during follow-up.

**Figure 11:**
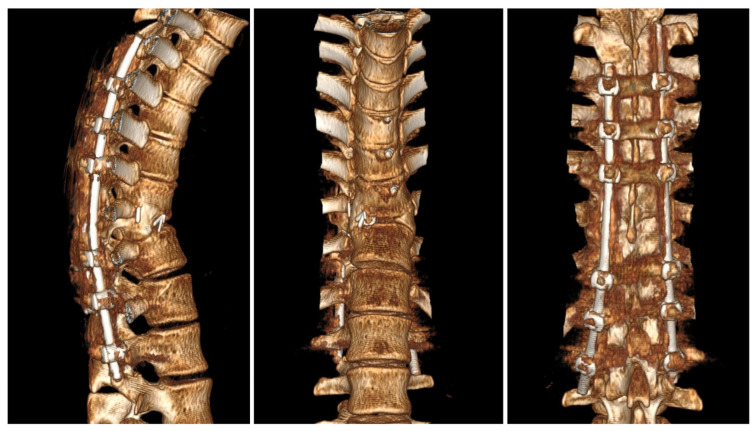
Three-year postoperative CT imaging demonstrating complete posterior spinal fusion of the operated spinal segments, with continuous trabecular bone formation across the fusion site and stable instrumentation. No signs of pseudarthrosis, implant failure, or loss of correction were identified.

Serial radiographic examinations demonstrated maintenance of deformity correction throughout the follow-up period. At three years after surgery, anteroposterior and lateral standing radiographs confirmed stable implant position, preservation of sagittal and coronal alignment, and no evidence of hardware failure, correction loss, or junctional complications (Figure 10). Computed tomography performed at the final follow-up demonstrated complete osseous fusion across the reconstructed spinal segment with continuous trabecular bone bridging both the anterior reconstruction and posterior fusion mass. No evidence of pseudarthrosis, cage subsidence, implant loosening, or correction loss was identified (Figure 11). Fusion was defined as continuous trabecular bone bridging across the anterior reconstruction and posterior fusion mass without implant loosening, hardware failure, or radiographic evidence of motion on serial imaging.

### 3.4. Functional and Radiographic Outcomes

Substantial improvements were observed in both radiographic parameters and patient-reported outcome measures (Table 1 and Table 2). The main thoracic Cobb angle improved from 32° preoperatively to 6° at final follow-up, corresponding to an 81.3% correction. Thoracic kyphosis (T2–T12) decreased from 78° to 63°, while focal kyphosis improved from 55° to 50°. Trunk height increased following reconstruction, with T1–T12 length increasing from 28 cm to 31 cm and T1–S1 length from 44 cm to 49 cm, reflecting restoration of spinal height and sagittal balance. Patient-reported outcomes also demonstrated marked clinical improvement. The SRS-22R total score increased from 3.85 preoperatively to 4.85 at final follow-up. Pain resolved completely, with the VAS score improving from 5/10 to 0/10, while disability substantially decreased, as reflected by an improvement in the Oswestry Disability Index (ODI) from 42 to 5. Throughout the entire follow-up period, no neurological deterioration, implant failure, loss of correction, or pseudarthrosis was observed.

Overall, the procedure achieved durable deformity correction, solid circumferential fusion, preservation of neurological function, excellent implant stability, and substantial improvement in patient-reported outcomes throughout the 36-month follow-up period.

## 4. Discussion

### 4.1. Technical Rationale of the Proposed Technique

The principal value of the described technique lies in integrating three technically demanding components—vertebral resection, anterior column reconstruction, and posterior stabilization—into a single-position lateral workflow. Conventional circumferential procedures for congenital thoracic kyphoscoliosis frequently require separate anterior and posterior stages, often with intraoperative repositioning, repeated preparation and draping, and interruption of the operative sequence [9,11,13,26,27,28,29,30,31,32,33,34,35].

In contrast, the present approach allows the entire procedure to be performed with the patient maintained in the lateral decubitus position, thereby preserving continuity of surgical exposure, fluoroscopic control, and intraoperative neuromonitoring. The lateral retropleural corridor was selected because it provides direct access to the thoracic vertebral body while avoiding a formal thoracotomy and limiting disruption of the posterior paraspinal musculature [19,20,22]. This exposure is particularly suitable for anterior release, vertebral body resection, endplate preparation, and cage placement. When combined with percutaneous pedicle screw fixation, it enables circumferential reconstruction while reducing the extent of posterior soft-tissue dissection. Similar lateral and retropleural techniques have been described for thoracic corpectomy, decompression, and interbody reconstruction; however, their application to congenital deformity correction with bilateral percutaneous instrumentation performed entirely in the lateral position remains rarely reported [19,20,21,22]. A further rationale for this strategy was the possibility of controlled, sequential deformity correction. Posterior fixation established initial mechanical control before vertebral resection, while gradual rod reduction and progressive cage expansion allowed correction to be distributed between the posterior and anterior columns. This sequence reduced reliance on a single corrective maneuver and facilitated restoration of both coronal and sagittal alignment under continuous fluoroscopic and neurophysiological surveillance. Rather than representing a collection of isolated technical maneuvers, the proposed procedure is based on a sequence of complementary biomechanical principles that work synergistically to achieve deformity correction, anterior column restoration, and immediate construct stability. These principles are summarized in Table 3.

Our described technique should therefore not be viewed merely as a less extensive access route, but as an integrated reconstructive concept. Its potential advantages arise from combining direct anterior access, percutaneous posterior fixation, controlled circumferential correction, and avoidance of intraoperative repositioning within one coordinated operative sequence. Nevertheless, these benefits remain theoretical in the absence of comparative data and should be interpreted cautiously given the single-case nature of the present report.

### 4.2. Comparison with Previously Reported Techniques

Although minimally invasive thoracic spinal surgery has evolved considerably over the past decade, its application to congenital spinal deformity correction remains limited. Most published reports have focused on thoracic corpectomy performed for degenerative disease, trauma, infection, or tumors rather than congenital kyphoscoliosis [19,20,21,22,23]. Consequently, evidence supporting minimally invasive deformity correction in this patient population is still scarce. Among the available reports, the method described by Ahmadian and Uribe [22] is probably the closest to the present technique. They described a mini-open lateral retropleural thoracic corpectomy using an expandable cage for the treatment of infectious vertebral destruction. However, their procedure was intended to achieve neural decompression and anterior column reconstruction rather than three-dimensional deformity correction. Furthermore, posterior percutaneous instrumentation was not performed in the lateral decubitus position, and the operation did not represent a single-position circumferential reconstruction. Similarly, Guiroy et al. [21] demonstrated the feasibility of single-position corpectomy with simultaneous posterior instrumentation for various thoracolumbar pathologies, primarily fractures and neoplastic conditions. Their study confirmed that maintaining the patient in a single position can improve the operative workflow and eliminate intraoperative repositioning. Nevertheless, congenital spinal deformities were not included, and the surgical objectives differed substantially from those of congenital kyphoscoliosis, where multiplanar deformity correction, restoration of spinal balance, and preservation of neurological function are equally important.

Recent anatomical and technical studies have also established the feasibility of lateral single-position surgery for lumbar reconstruction. Lateral-position anterior lumbar interbody fusion (ALIF), lateral lumbar interbody fusion (LLIF), and percutaneous pedicle screw fixation performed without repositioning have been shown to reduce operative workflow interruptions while maintaining satisfactory implant placement [24,25,36,37,38]. These studies provide an important technical foundation for single-position spinal surgery but are confined to the lumbar spine and do not address vertebral resection or correction of congenital thoracic deformities. In contrast, the literature addressing congenital kyphosis and kyphoscoliosis remains dominated by extensive posterior osteotomies, vertebral column resection (VCR), pedicle subtraction osteotomy (PSO), and combined anterior–posterior procedures [18,39,40,41,42,43,44,45,46,47,48,49,50,51,52,53,54]. These techniques consistently achieve substantial deformity correction but are associated with prolonged operative time, considerable blood loss, neurological risk, and procedure-related morbidity, particularly in severe rigid deformities [18,39,40,44,45,47,50,53,54]. Consequently, there has been increasing interest in less invasive strategies capable of achieving satisfactory correction while reducing surgical morbidity.

The present technique should therefore be viewed as complementary to, rather than competitive with, established reconstructive procedures. It is not intended to replace posterior VCR or other complex osteotomies in patients with extremely rigid multiplanar deformities. Instead, it expands the surgical armamentarium by offering a reproducible single-position strategy for carefully selected patients in whom direct lateral access permits vertebral resection, anterior column reconstruction, and percutaneous posterior stabilization through a less invasive operative corridor. To the best of our knowledge, this is the first published report describing correction of congenital thoracic kyphoscoliosis using a lateral retropleural vertebral resection combined with bilateral percutaneous pedicle screw fixation performed entirely in the lateral decubitus position. The principal novelty of the technique therefore lies not only in the retropleural exposure itself, but in the integration of vertebral resection, circumferential reconstruction, and definitive posterior fixation into a single uninterrupted operative workflow.

### 4.3. Technical Advantages and Practical Considerations

Based on our operative experience, the most important technical recommendations and potential pitfalls of this procedure are summarized in Table 4, providing a practical reference for surgeons adopting this technique.

Beyond the novelty of performing circumferential reconstruction without intraoperative repositioning, the present technique offers several practical technical advantages. Maintaining the patient in a stable lateral decubitus position throughout the procedure eliminates repeated positioning, redraping, and re-registration of fluoroscopic landmarks, thereby simplifying the operative workflow and preserving uninterrupted access to both the anterior and posterior columns. Continuous positioning also allows uninterrupted intraoperative neuromonitoring and facilitates communication between the surgical and anesthesia teams during critical stages of deformity correction [21,24,25,36,37,38].

The retropleural approach provides direct access to the anterior thoracic spine while avoiding formal thoracotomy. Preservation of the extrapleural plane minimizes disruption of the chest wall musculature and reduces manipulation of intrathoracic structures compared with conventional transthoracic approaches [20,22]. Although inadvertent pleural violation remains a recognized risk, the retropleural corridor generally permits adequate vertebral exposure while potentially reducing the pulmonary morbidity and postoperative pain associated with open thoracic procedures [20,22]. An important technical aspect of the present procedure is the combination of anterior vertebral reconstruction with percutaneous posterior fixation. Placement of pedicle screws before vertebral resection establishes provisional spinal stability and provides secure anchor points for controlled correction. Progressive rod reduction followed by gradual expansion of the anterior cage distributes corrective forces between the anterior and posterior columns, avoiding abrupt correction maneuvers that may increase stress on the spinal cord or instrumentation. From a biomechanical perspective, circumferential load sharing achieved by the expandable cage and posterior pedicle screw construct enhances construct stability while reducing stress concentration on the posterior rods [30,39,40,43,55,56]. Percutaneous pedicle screw placement in the lateral decubitus position represents one of the most technically demanding components of the procedure. Congenital vertebral anomalies frequently distort pedicle morphology and anatomical landmarks, making meticulous preoperative imaging analysis and precise fluoroscopic guidance mandatory. The tunnel-view fluoroscopic technique has been shown to improve visualization of the pedicle trajectory during lateral instrumentation and may enhance the safety of screw placement in single-position surgery [24]. In centers with access to advanced technology, intraoperative three-dimensional navigation or robotic assistance may further improve screw accuracy while simplifying the workflow of lateral-position instrumentation.

Despite these potential advantages, the described technique has a substantial learning curve. Successful execution requires familiarity with retropleural anatomy, minimally invasive thoracic exposure, percutaneous spinal instrumentation, deformity correction principles, and anterior column reconstruction. Consequently, this approach should currently be reserved for specialized spine centers with experience in both complex spinal deformity surgery and minimally invasive thoracic procedures. Appropriate patient selection remains essential, and the technique should not be considered a universal alternative to conventional posterior osteotomies or vertebral column resection. Finally, it should be emphasized that the proposed strategy is intended to reduce surgical exposure rather than compromise the fundamental principles of deformity correction. Adequate visualization, meticulous neural decompression, stable circumferential reconstruction, and restoration of global spinal alignment remain the primary surgical objectives irrespective of the chosen surgical corridor.

### 4.4. Indications and Patient Selection

Appropriate patient selection is probably the most important determinant of successful implementation of the proposed technique. The present approach should not be considered a universal alternative to conventional posterior osteotomies or vertebral column resection. Rather, it should be reserved for carefully selected patients in whom the anatomical characteristics of the deformity permit safe lateral retropleural access and circumferential reconstruction through a single-position workflow. Factors that should be considered during surgical planning include deformity morphology, vertebral anatomy, spinal flexibility, neurological status, pulmonary reserve, and the surgeon’s experience with minimally invasive thoracic procedures. Based on the present experience and the available literature, the proposed indications and limitations of this technique are summarized in Table 5 [17,18,39,40,41,42,43,44,45,46,47,48,49,50,51,52,53,54,57,58].

The proposed indications are based on the present technical experience and the currently available literature [17,18,39,40,41,42,43,44,45,46,47,48,49,50,51,52,53,54,57,58].

### 4.5. Neurological Safety and Perioperative Risk Management

Neurological preservation remains the primary objective of corrective surgery for congenital spinal deformities. Procedures involving vertebral resection, anterior column reconstruction, and multiplanar deformity correction expose the spinal cord to simultaneous mechanical, vascular, and traction-related stress. Consequently, neurological safety depends not only on the technical accuracy of the osteotomy or instrumentation but also on meticulous perioperative planning, controlled correction maneuvers, and continuous intraoperative monitoring [57,59,60,61,62]. Large clinical series have consistently demonstrated that congenital spinal deformities, vertebral column resection, severe angular kyphosis, extensive three-column osteotomies, prolonged operative time, and excessive blood loss represent major risk factors for intraoperative neuromonitoring (IONM) alerts and postoperative neurological deficits [18,57,61]. Contemporary management therefore emphasizes immediate identification of neurophysiological signal deterioration together with a structured multidisciplinary response aimed at restoring spinal cord perfusion, excluding mechanical compression, and reversing potentially harmful correction maneuvers [61,62].

In the present case, neurological preservation was likely facilitated by several complementary technical principles rather than by any single maneuver. First, provisional posterior fixation established mechanical stability before vertebral resection. Second, deformity correction was performed gradually through sequential rod reduction and controlled expansion of the anterior cage instead of forceful correction at a single stage. Third, uninterrupted multimodal neuromonitoring provided continuous functional assessment of the spinal cord throughout the procedure. Finally, close coordination between the surgical and anesthesia teams allowed maintenance of adequate spinal cord perfusion during the most demanding stages of reconstruction. The only perioperative complication encountered was postoperative pneumothorax secondary to pleural violation during retropleural exposure. Although the retropleural approach is specifically designed to avoid entering the pleural cavity, pleural injury remains a recognized complication, particularly in patients with congenital deformities, in whom normal thoracic anatomy is frequently distorted [20,22]. Prompt postoperative recognition and tube thoracostomy resulted in complete pulmonary re-expansion without delaying neurological recovery or functional rehabilitation. The occurrence of this complication also highlights an important practical aspect of the proposed technique. A less invasive surgical corridor should not be interpreted as a risk-free procedure. Rather, successful implementation depends on thorough preoperative anatomical assessment, careful retropleural dissection, meticulous fluoroscopic control, and immediate recognition and management of procedure-specific complications. In experienced hands, these risks appear manageable and should be weighed against the potential benefits of avoiding formal thoracotomy, extensive posterior muscle dissection, and intraoperative repositioning.

Overall, this case illustrates that complex congenital deformity correction can be performed safely through a single-position lateral workflow when meticulous surgical technique is combined with structured perioperative risk management, continuous neuromonitoring, and disciplined stepwise correction. Nevertheless, broader clinical experience will be required to determine whether these theoretical advantages translate into lower neurological morbidity compared with conventional open reconstructive procedures.

### 4.6. Clinical Implications and Future Perspectives

The present report should not be interpreted as proposing a replacement for established reconstructive techniques used in congenital spinal deformity surgery. Posterior vertebral column resection, pedicle subtraction osteotomy, posterior hemivertebra resection, growth-friendly systems, and halo-gravity traction remain indispensable components of the contemporary surgical armamentarium, with treatment strategies tailored to patient age, deformity morphology, flexibility, neurological status, and skeletal maturity [17,18,39,40,41,42,43,44,45,46,47,48,49,50,51,52,53,54,57,58,63,64,65,66,67,68,69,70,71,72,73]. Rather, the technique described here expands the spectrum of available surgical options for carefully selected patients with thoracic congenital deformities. In cases where direct lateral access is anatomically feasible, combining retropleural vertebral resection, anterior column reconstruction, and percutaneous posterior stabilization within a single-position workflow may simplify the operative sequence while preserving the fundamental principles of deformity correction. Importantly, the objective of this approach is not to maximize the magnitude of correction but to achieve safe circumferential reconstruction with stable spinal alignment, solid fusion, and preservation of neurological function.

From a technical perspective, the concept aligns with the broader evolution of spinal surgery toward procedure-specific optimization rather than maximal surgical exposure. Advances in intraoperative imaging, three-dimensional navigation, robotic assistance, expandable interbody implants, and minimally invasive instrumentation continue to expand the indications for less invasive reconstructive procedures [22,23,24,25]. As these technologies become more widely available, the feasibility and reproducibility of single-position circumferential reconstruction may further improve, particularly in specialized deformity centers. Nevertheless, wider clinical adoption should proceed cautiously. The present technique demands advanced familiarity with thoracic retropleural anatomy, minimally invasive spinal exposure, complex deformity correction, and percutaneous instrumentation. Appropriate patient selection, comprehensive preoperative imaging, and meticulous surgical planning remain essential prerequisites. Future technological developments should therefore complement—not replace—sound surgical judgment and adherence to established principles of spinal deformity correction.

Taken together, the advantages and limitations summarized in Table 6 suggest that this technique should currently be regarded as a valuable addition to the armamentarium of complex spinal deformity surgery rather than a replacement for established reconstructive procedures.

Prospective multicenter studies comparing this strategy with conventional open circumferential procedures will be necessary to determine whether the proposed workflow offers measurable advantages regarding operative time, blood loss, pulmonary morbidity, postoperative recovery, implant accuracy, fusion rates, patient-reported outcomes, and long-term maintenance of deformity correction. Such investigations will ultimately define the appropriate role of this technique within modern congenital spine surgery.

### 4.7. Limitations

Several limitations should be acknowledged. First, this report describes the technical application of the proposed workflow in a single patient and therefore does not permit conclusions regarding superiority, reproducibility, or comparative effectiveness. Second, congenital thoracic kyphoscoliosis encompasses a heterogeneous group of deformities with considerable anatomical variability; consequently, the described technique is unlikely to be applicable to all patients requiring complex spinal reconstruction. Third, although the patient demonstrated excellent clinical and radiographic outcomes at 36 months, longer follow-up is necessary to confirm maintenance of correction, implant durability, and long-term spinal growth and balance where applicable.

An additional limitation relates to the technical demands of the procedure itself. Successful implementation requires experience with retropleural thoracic exposure, minimally invasive instrumentation, complex deformity correction, and management of potential thoracic complications. The learning curve may therefore limit early adoption outside specialized spine centers. Finally, the absence of a comparison with conventional open circumferential reconstruction precludes assessment of whether the proposed strategy provides measurable reductions in operative morbidity, pulmonary complications, or recovery time.

Despite these limitations, the present Technical Note demonstrates the feasibility of integrating vertebral resection, anterior column reconstruction, and bilateral percutaneous pedicle screw fixation into a single-position lateral workflow. We believe that this report provides a reproducible technical framework that may serve as a foundation for future clinical studies evaluating the safety, reproducibility, and broader applicability of this operative strategy.

## 5. Conclusions

This Technical Note presents a novel single-position strategy for congenital thoracic kyphoscoliosis correction that integrates lateral retropleural vertebral resection, anterior column reconstruction, and bilateral percutaneous pedicle screw fixation within a continuous operative workflow. In the present case, the technique achieved durable deformity correction, solid spinal reconstruction, and preservation of neurological function without implant-related complications. Although broader validation is required, this approach may represent a valuable addition to the surgical armamentarium for selected congenital thoracic deformities and provides a reproducible technical framework for further clinical investigation.

## Figures and Tables

**Table 1:** Patient-reported outcome measures before surgery and at final follow-up.

Questionnaire Outcomes	Patient Outcome
SRS-22R total score, preoperatively	3.85
SRS-22R total score, at the final follow-up	4.85
VAS score, preoperatively	5/10
VAS score, at the final follow-up	0/10
ODI score, preoperatively	42
ODI score, at the final follow-up	5

**Table 2:** Pre- and postoperative parameters for treated patient.

Parameter	Preoperative	Final Follow-Up
T1–T12 length (cm)	28	31
T1–S1 length (cm)	44	49
Main thoracic Cobb (°)	32	6
T2–T5 kyphosis (°)	19	12
T5–T12 kyphosis (°)	60	47
Thoracic kyphosis T2–T12 (°)	78	63
Focal kyphosis (°)	55	50
Lumbar lordosis (°)	81	65

**Table 3:** Biomechanical rationale of the proposed single-position reconstruction strategy.

Biomechanical Principle	Surgical Implementation	Expected Effect
Temporary spinal stability	Long-segment percutaneous pedicle screw fixation (T7–L1) performed before vertebral resection	Maintains spinal stability throughout corpectomy and provides a stable foundation for controlled deformity correction.
Removal of the deformity apex	Piecemeal T10 hemicorpectomy while preserving the posterior vertebral cortex until completion of the resection	Eliminates the structural driver of the deformity while protecting the spinal cord during reconstruction.
Anterior column restoration	Expandable titanium cage inserted through the retropleural corridor and gradually expanded	Restores anterior column height, re-establishes sagittal alignment, and improves physiological load transmission.
Controlled deformity correction	Progressive rod reduction followed by gradual cage expansion under fluoroscopic guidance	Enables simultaneous correction of kyphosis and scoliosis while minimizing excessive forces on the spinal cord and instrumentation.
Circumferential load sharing	Combined anterior column support and posterior pedicle screw fixation	Distributes physiological loads between the anterior and posterior spinal columns, reducing stress concentration within the posterior construct.
Long-term biological stability	Circumferential fusion using locally harvested autologous bone graft supplemented with bone substitute	Promotes solid arthrodesis, minimizes the risk of pseudarthrosis, and improves long-term construct durability.
Single-position workflow	Posterior instrumentation, vertebral resection, anterior reconstruction, and final fixation performed entirely in the lateral decubitus position	Eliminates intraoperative repositioning, maintains uninterrupted neuromonitoring, and streamlines the surgical workflow.

**Table 4:** Technical pearls, pitfalls, and intraoperative considerations for single-position lateral retropleural reconstruction.

Operative Step	Technical Pearls	Technical Pitfalls
Patient positioning	Achieve a true lateral decubitus position before skin preparation. Pad all pressure points and ensure unrestricted fluoroscopic access throughout the procedure.	Rotational malposition may compromise fluoroscopic orientation, pedicle screw trajectories, and cage positioning.
Fluoroscopic setup	Confirm true AP and lateral projections before every critical step. Minor table adjustments are preferable to repeated C-arm repositioning.	Poor fluoroscopic alignment may result in inaccurate implant placement or inadequate deformity correction.
Percutaneous pedicle screw insertion	Insert all pedicle screws before beginning the lateral exposure. Place temporary rods only after confirming satisfactory screw position.	Instrumentation after corpectomy may reduce construct stability and increase technical difficulty, particularly on the dependent side.
Retropleural exposure	Maintain meticulous subperiosteal rib dissection and preserve the extrapleural plane whenever possible. Use blunt dissection to minimize pleural mobilization.	Excessive pleural dissection increases the risk of pleural violation and postoperative pneumothorax.
Vertebral resection	Preserve the posterior vertebral cortex until completion of the anterior resection to maintain temporary stability and protect the spinal cord. Perform piecemeal vertebral removal under direct microscopic visualization.	Aggressive posterior wall resection may increase the risk of neurological injury or uncontrolled instability.
Anterior column reconstruction	Prepare both endplates carefully before cage insertion. Pack the expandable cage with autologous bone graft supplemented with bone substitute. Expand the cage gradually under continuous fluoroscopic control.	Inadequate endplate preparation or asymmetric cage expansion may lead to implant subsidence, malalignment, or residual deformity.
Deformity correction	Perform gradual rod reduction before final cage expansion. Confirm coronal and sagittal correction fluoroscopically before tightening the locking caps.	Rapid correction or premature locking may produce excessive stress on the construct and limit final correction.
Final construct assessment	Verify implant position in both AP and lateral projections and confirm stable neuromonitoring before wound closure.	Failure to identify implant malposition or residual imbalance before closure may necessitate early revision surgery.

**Table 5:** Proposed indications and technical limitations of the single-position lateral retropleural approach for congenital thoracic kyphoscoliosis.

Potential Indications	Relative Contraindications/Technical Limitations
Selected congenital thoracic kyphosis or kyphoscoliosis requiring anterior column reconstruction	Extremely severe rigid multiplanar deformities requiring extensive posterior osteotomies
Progressive deformity with pain, imbalance, or evolving neurological risk	Extensive pleural adhesions or previous thoracic surgery
Adequate lateral access to the pathological vertebra	Inability to obtain safe fluoroscopic visualization
Need for circumferential reconstruction without formal thoracotomy	Complex vascular or visceral anatomical anomalies
Patients in whom minimizing posterior muscle dissection may be advantageous	Marked vertebral dysplasia precluding safe percutaneous screw placement
Availability of experienced multidisciplinary spine team	Limited experience with retropleural and minimally invasive thoracic surgery

**Table 6:** Potential advantages and current limitations of the proposed single-position reconstruction strategy.

Potential Advantages	Current Limitations
Single-position surgery without patient repositioning	Experience currently limited to selected patients
Simultaneous anterior reconstruction and posterior stabilization	Technically demanding procedure with a significant learning curve
Reduced posterior muscle dissection through percutaneous instrumentation	Requires advanced fluoroscopic guidance and familiarity with minimally invasive spinal techniques
Retropleural access avoids formal thoracotomy	Pleural violation remains possible despite retropleural dissection
Continuous neuromonitoring without interruption caused by repositioning	Lack of comparative clinical studies versus conventional techniques
Direct lateral access to the vertebral body with excellent visualization	Applicability to severe multiplanar deformities requires further investigation
Potential reduction in operative workflow interruptions	Long-term multicenter validation is still needed

## Data Availability

Data are contained within the article.
